# Supplementary material for: Activity-dependent remodeling of muscle architecture during distinct locomotor behaviors in Caenorhabditis elegans
Source: Biol Open. 2026 Jan 26;15(1):bio062371. doi: 10.1242/bio.062371 (PMC12869499; doi:10.1242/bio.062371)
Supplement: Supplementary information [file biolopen-15-062371-s1.pdf]

```
# LIBRARIES
suppressPackageStartupMessages({
  if (!requireNamespace("emmeans", quietly = TRUE)) {
    install.packages("emmeans", repos = "https://cloud.r-project.org")
  }
  library(emmeans)
  library(ggplot2)
})
suppressPackageStartupMessages({
  library(tidyverse)
  library(readxl)
  library(openxlsx)
  library(lme4)
  library(lmerTest)
  library(sandwich)
  library(lmtest)
})
emmeans::emm_options(lmer.df = "satterthwaite")
INPUT_XLSX  <- "/Users/adinafazyl/Desktop/Andres/Supplementary1.xlsx"
SHEET_INDEX <- 1
OUTPUT_XLSX <- "LMM_Report_NEWFILE_ROBUST_R_new.xlsx"
IQR_K <- 1.5
MIN_NONMISSING <- 20
to_numeric_eu <- function(x) {
  if (is.numeric(x)) return(x)
  val <- suppressWarnings(as.numeric(x))
  if (any(!is.na(val) & !is.na(x))) return(val)
  val <- suppressWarnings(as.numeric(gsub(",", ".", x)))
  return(val)
}
standardize_id_cols <- function(df) {
  df <- df %>%
    rename_with(~ case_when(
      . == "Region"  ~ "region",
      . == "WormID"  ~ "worm",
      . == "Regimen" ~ "condition",
      TRUE ~ .
    ))
  if ("region" %in% names(df)) {
    df$region <- stringr::str_to_title(stringr::str_trim(as.character(df$region)))
    region_map <- c("Head"="Head","Anterior"="Head",
                    "Mid"="Mid","Medial"="Mid",
                    "Tail"="Tail","Posterior"="Tail")
    df$region <- ifelse(df$region %in% names(region_map),
                        unname(region_map[df$region]), df$region)
  }
  if ("condition" %in% names(df)) {
    df$condition <- stringr::str_to_title(stringr::str_trim(as.character(df$condition)))
```

Biology Open • Supplementary information

```
    cond_map <- c("Crawl"="Crawl","Swim"="Swim")
    df$condition <- ifelse(df$condition %in% names(cond_map),
                           unname(cond_map[df$condition]), df$condition)
  }
  if ("worm" %in% names(df)) {
    df$worm <- stringr::str_trim(as.character(df$worm))
  }
  df
}
existing_names <- function(wb) {
  if (is.null(wb$sheet_names)) character(0) else wb$sheet_names
}
compute_icc <- function(df, dep, group = "worm") {
  df_clean <- df %>% dplyr::select(dplyr::all_of(c(dep, group))) %>% tidyr::drop_na()
  if (nrow(df_clean) == 0) return(0)
  k <- dplyr::n_distinct(df_clean[[group]])
  N <- nrow(df_clean)
  if (k <= 1 || N <= k) return(0)
  tryCatch({
    f <- as.formula(paste0("`", dep, "` ~ 1 + (1 | ", group, ")"))
    m0 <- lme4::lmer(f, data = df_clean, REML = TRUE)
    vc <- as.data.frame(VarCorr(m0))
    tau2 <- vc[vc$grp == group, "vcov"]
    sigma2 <- vc[vc$grp == "Residual", "vcov"]
    if (length(tau2) == 0 || length(sigma2) == 0) return(0)
    icc <- tau2 / (tau2 + sigma2)
    as.numeric(icc)
  }, error = function(e) 0)
}
# Simple-effects helper (tries LMM w/ KR → Satt fallback; else OLS)
fit_and_emm <- function(dat_use, dep, group_col = "worm") {
  dat_use <- dat_use %>% mutate(condition=factor(condition), region=factor(region))
  icc_val <- compute_icc(dat_use, dep, group_col)
  lmm_ok <- FALSE
  emm <- NULL
  try({
    lmm_fit <- suppressWarnings(lmerTest::lmer(
      as.formula(paste0("`", dep, "` ~ condition * region + (1|", group_col,")")),
      data = dat_use, REML = TRUE,
      control = lmerControl(optimizer="bobyqa", check.conv.singular="ignore")
    ))
    vc <- as.data.frame(VarCorr(lmm_fit))
    rand_var <- vc[vc$grp==group_col,"vcov"]; rand_var <- if (length(rand_var)) rand_var else NA_real_
    if (!isSingular(lmm_fit, tol=1e-4) && !is.na(rand_var) && rand_var >= 1e-8 && icc_val >= 0.01) {
      ok <- FALSE

      try({ emmeans::emm_options(lmer.df="kenward-roger"); emm <- emmeans(lmm_fit, ~ condition |
region); ok <- TRUE }, silent=TRUE)
```

Biology Open • Supplementary information

```
      if (!ok) { emmeans::emm_options(lmer.df="satterthwaite"); emm <- emmeans(lmm_fit, ~ condition |
region) }
      lmm_ok <- TRUE
    }
  }, silent = TRUE)
  if (lmm_ok) return(list(kind="lmm", emm=emm))
  ols_fit <- lm(as.formula(paste0("`", dep, "` ~ condition * region")), data=dat_use)
  emm <- emmeans(ols_fit, ~ condition | region)
  list(kind="ols", emm=emm)
}
try_mixed_then_robust_ols <- function(df, dep, group_col = "worm") {
  df <- df %>% mutate(condition=factor(condition), region=factor(region))
  fixed_f <- paste0("`", dep, "` ~ condition * region")
  mixed_f <- as.formula(paste0(fixed_f, " + (1 | ", group_col, ")"))
  ols_f   <- as.formula(fixed_f)
  model_type <- "MixedLM(KR/Satt)"
  diag <- list(warning = NA_character_, rand_var = NA_real_, icc = NA_real_, df_method =
NA_character_)
  results_rows <- list()
  icc_val <- compute_icc(df, dep, group_col)
  diag$icc <- icc_val
  tryCatch({
    lmm_fit <- lmerTest::lmer(
      mixed_f, data = df, REML = TRUE,
      control = lmerControl(optimizer = "bobyqa")
    )
    vc <- as.data.frame(VarCorr(lmm_fit))
    rand_var <- vc[vc$grp == group_col, "vcov"]; rand_var <- if (length(rand_var)) rand_var else NA_real_
    diag$rand_var <- rand_var
    if (isSingular(lmm_fit, tol = 1e-4) || is.na(rand_var) || rand_var < 1e-8 || icc_val < 0.01) {
      stop("Fallback condition met (Singular, boundary, or low ICC).")
    }
    summ <- tryCatch(
      summary(lmm_fit, ddf = "Kenward-Roger"),
      error = function(e) summary(lmm_fit, ddf = "Satterthwaite")
    )
    coefs <- summ$coefficients
    have_df <- "df" %in% colnames(coefs)
    dfs <- if (have_df) coefs[, "df"] else Inf
    tcrit <- if (have_df) qt(0.975, dfs) else rep(1.96, nrow(coefs))
    ci_lo <- coefs[, "Estimate"] - tcrit * coefs[, "Std. Error"]
    ci_hi <- coefs[, "Estimate"] + tcrit * coefs[, "Std. Error"]
    df_method <- if (!is.null(summ$methTitle) && grepl("Kenward", summ$methTitle)) "Kenward-Roger"
else "Satterthwaite"
    diag$df_method <- df_method
    for (i in seq_len(nrow(coefs))) {
      results_rows[[length(results_rows) + 1]] <- tibble(
        Dependent = dep,
        Param     = rownames(coefs)[i],
```

Biology Open • Supplementary information

```
        Estimate  = coefs[i, "Estimate"],
        CI95_L    = ci_lo[i],
        CI95_U    = ci_hi[i],
        p_value   = coefs[i, "Pr(>|t|)"],
        Model     = model_type,
        RandVar   = rand_var,
        ICC_proxy = icc_val,
        N_used    = nobs(lmm_fit),
        DF        = if (have_df) dfs[i] else NA_real_,
        DF_method = df_method
      )
    }
  }, error = function(e) {
    diag$warning  <<- if (grepl("Fallback condition met", e$message)) "Boundary/Singular/LowICC" else
paste0("LMM error: ", e$message)
    model_type    <<- "OLS-CR"
    ols_fit <- lm(ols_f, data = df)
    vcov_cr <- sandwich::vcovCL(ols_fit, cluster = df[[group_col]], type = "HC1")
    ct <- lmtest::coeftest(ols_fit, vcov. = vcov_cr)
    estimates <- ct[, "Estimate"]
    stderrs   <- ct[, "Std. Error"]
    ci_lo <- estimates - 1.96 * stderrs
    ci_hi <- estimates + 1.96 * stderrs
    pvals <- ct[, "Pr(>|t|)"]
    for (i in seq_len(nrow(ct))) {
      results_rows[[length(results_rows) + 1]] <<- tibble(
        Dependent = dep,
        Param     = rownames(ct)[i],
        Estimate  = estimates[i],
        CI95_L    = ci_lo[i],
        CI95_U    = ci_hi[i],
        p_value   = pvals[i],
        Model     = model_type,
        RandVar   = NA_real_,
        ICC_proxy = icc_val,
        N_used    = nobs(ols_fit),
        DF        = NA_real_,
        DF_method = "ClusterRobustHC1"
      )
    }
  })
  list(model_type = model_type, rows = dplyr::bind_rows(results_rows), diag = diag)
}
cat("Reading ", INPUT_XLSX, " (sheet ", SHEET_INDEX, ")...\n", sep = "")
df0 <- readxl::read_excel(INPUT_XLSX, sheet = SHEET_INDEX, .name_repair = "minimal")
df1 <- standardize_id_cols(df0)
id_cols <- c("region", "worm", "condition")
all_cols <- names(df1)
dep_candidates <- setdiff(all_cols, id_cols)
```

Biology Open • Supplementary information

```
df <- df1
for (col in dep_candidates) {
  df[[col]] <- to_numeric_eu(df[[col]])
}
dep_vars <- c()
for (v in dep_candidates) {
  if (is.numeric(df[[v]])) {
    n_not_na <- sum(!is.na(df[[v]]))
    n_unique <- dplyr::n_distinct(na.omit(df[[v]]))
    if (n_not_na >= MIN_NONMISSING && n_unique >= 2) dep_vars <- c(dep_vars, v)
  }
}
wb <- openxlsx::createWorkbook()
openxlsx::addWorksheet(wb, "INDEX")
index_df <- tibble(
  Metric = c("N_rows_raw", "N_dep_candidates", "Dep_Vars_Selected"),
  Value  = c(as.character(nrow(df)), as.character(length(dep_candidates)), paste(dep_vars, collapse = ",
"))
)
openxlsx::writeData(wb, "INDEX", index_df)
openxlsx::addWorksheet(wb, "Raw_Data")
openxlsx::writeData(wb, "Raw_Data", df)
all_outlier_logs <- list()
all_iqr_stats    <- list()
all_model_rows   <- list()
all_diag_rows    <- list()
simple_list <- list()
plot_list   <- list()
cat("Starting per-variable analysis...\n")
for (dep in dep_vars) {
  dat_v <- df %>%
    dplyr::select(dplyr::all_of(c(id_cols, dep))) %>%
    dplyr::filter(!is.na(.data[[dep]]))
  grp_min_n <- 8
  bounds <- dat_v %>%
    dplyr::group_by(condition, region) %>%
    dplyr::summarise(
      n_grp = dplyr::n(),
      Q1    = ifelse(n_grp >= grp_min_n, stats::quantile(.data[[dep]], 0.25, na.rm = TRUE), NA_real_),
      Q3    = ifelse(n_grp >= grp_min_n, stats::quantile(.data[[dep]], 0.75, na.rm = TRUE), NA_real_),
      IQRv  = ifelse(n_grp >= grp_min_n, Q3 - Q1, NA_real_),
      MADv  = ifelse(n_grp >= grp_min_n, stats::mad(.data[[dep]], constant = 1.4826, na.rm = TRUE),
NA_real_),
      medv  = ifelse(n_grp >= grp_min_n, stats::median(.data[[dep]], na.rm = TRUE), NA_real_),
      use_mad = ifelse(!is.na(IQRv) & IQRv == 0 & !is.na(MADv) & MADv > 0, TRUE, FALSE),
      lo = dplyr::case_when(
        n_grp < grp_min_n ~ NA_real_,
        use_mad           ~ medv - IQR_K * 1.349 * MADv,
        TRUE              ~ Q1   - IQR_K * IQRv
```

```
      ),
      hi = dplyr::case_when(
        n_grp < grp_min_n ~ NA_real_,
        use_mad           ~ medv + IQR_K * 1.349 * MADv,
        TRUE              ~ Q3   + IQR_K * IQRv
      ),
      .groups = "drop"
    )
  dat_with_bounds <- dat_v %>%
    dplyr::left_join(bounds, by = c("condition","region"))
  is_outlier_grp <- !is.na(dat_with_bounds$lo) & !is.na(dat_with_bounds$hi) &
                    ((dat_with_bounds[[dep]] < dat_with_bounds$lo) |
                     (dat_with_bounds[[dep]] > dat_with_bounds$hi))
  if (any(is_outlier_grp, na.rm = TRUE)) {
    all_outlier_logs[[length(all_outlier_logs) + 1]] <- dat_with_bounds %>%
      dplyr::mutate(value = .data[[dep]]) %>%
      dplyr::filter(is_outlier_grp) %>%
      dplyr::transmute(
        Dependent = dep, condition, region,
        value, lower = lo, upper = hi
      )
  }
  all_iqr_stats[[length(all_iqr_stats) + 1]] <- dat_with_bounds %>%
    dplyr::mutate(is_outlier = is_outlier_grp) %>%
    dplyr::group_by(Dependent = dep, condition, region) %>%
    dplyr::summarise(
      n_total   = dplyr::n(),
      n_outliers= sum(is_outlier, na.rm = TRUE),
      lower     = dplyr::first(lo),
      upper     = dplyr::first(hi),
      .groups   = "drop"
    )
  dat_use <- dat_with_bounds %>%
    dplyr::filter(is.na(lo) | is.na(hi) | (.data[[dep]] >= lo & .data[[dep]] <= hi)) %>%
    dplyr::select(dplyr::all_of(c(id_cols, dep))) %>%
    tidyr::drop_na() %>%
    droplevels()
  if (dplyr::n_distinct(dat_use$worm) >= 2 &&
      dplyr::n_distinct(dat_use$condition) >= 2 &&
      dplyr::n_distinct(dat_use$region) >= 2) {
    fit <- fit_and_emm(dat_use, dep, group_col="worm")
    contr <- contrast(fit$emm, method = list("Swim - Crawl" = c(-1, 1)), by="region") %>%
      summary(infer = c(TRUE, TRUE)) %>%
      as.data.frame() %>%
      dplyr::mutate(Dependent = dep) %>%
      dplyr::select(Dependent, region, estimate, SE, df, t.ratio, p.value, lower.CL, upper.CL)
    simple_list[[length(simple_list)+1]] <- contr

    summ <- dat_use %>%
```

Biology Open • Supplementary information

```
    dplyr::group_by(region, condition) %>%
    dplyr::summarise(mean = mean(.data[[dep]]), sd = sd(.data[[dep]]),
                     N=dplyr::n(), sem = sd/sqrt(N), .groups="drop")
  lab <- contr %>%
    dplyr::mutate(
      y = sapply(region, function(r){
        max(summ$mean[summ$region==r] + summ$sem[summ$region==r]) * 1.05
      }),
      label = dplyr::case_when(
        p.value < 0.001 ~ "***",
        p.value < 0.01  ~ "**",
        p.value < 0.05  ~ "*",
        TRUE            ~ "ns"
      )
    )
  p <- ggplot(summ, aes(x=region, y=mean, fill=condition)) +
    geom_col(position=position_dodge(width=0.6), width=0.55) +
    geom_errorbar(aes(ymin=mean-sem, ymax=mean+sem),
                  position=position_dodge(width=0.6), width=0.2) +
    geom_text(data=lab, aes(x=region, y=y, label=label),
              inherit.aes=FALSE, vjust=0) +
    labs(title=paste0(dep, ": Swim vs Crawl by region"),
         y=dep, x="Region") +
    theme_classic()
  plot_list[[dep]] <- p
}
safe_dep_name <- substr(dep, 1, 15)
summ_sheet <- paste0(safe_dep_name, "__GrpSum")
count <- 1
while (summ_sheet %in% existing_names(wb)) {
  summ_sheet <- paste0(substr(paste0(safe_dep_name, "__GrpSum"), 1, 25), "_", count)
  count <- count + 1
}
if (nrow(dat_use) > 0) {
  grp_sum <- dat_use %>%
    dplyr::group_by(region, condition) %>%
    dplyr::summarise(
      N   = dplyr::n(),
      mean= mean(.data[[dep]]),
      sd  = sd(.data[[dep]]),
      .groups = "drop"
    ) %>%
    dplyr::mutate(sem = sd / sqrt(N)) %>%
    dplyr::rename_with(~ paste0(dep, "_", .), .cols = c("mean", "sd", "sem"))
  openxlsx::addWorksheet(wb, summ_sheet)
  openxlsx::writeData(wb, summ_sheet, grp_sum)
}
n_w <- dplyr::n_distinct(dat_use$worm)
n_c <- dplyr::n_distinct(dat_use$condition)
```

Biology Open • Supplementary information

```
  n_r <- dplyr::n_distinct(dat_use$region)
  if (n_w < 2 || n_c < 2 || n_r < 2) {
    all_diag_rows[[length(all_diag_rows) + 1]] <- tibble(
      Dependent = dep, Model = "SKIPPED", Warning = "Not enough levels",
      ICC_proxy = NA_real_, RandVar = NA_real_, DF_method = NA_character_
    )
    next
  }
  res <- try_mixed_then_robust_ols(dat_use, dep, group_col = "worm")
  all_model_rows[[length(all_model_rows) + 1]] <- res$rows
  all_diag_rows[[length(all_diag_rows) + 1]] <- tibble(
    Dependent = dep,
    Model     = res$model_type,
    ICC_proxy = res$diag$icc,
    RandVar   = res$diag$rand_var,
    DF_method = if (is.null(res$diag$df_method)) NA_character_ else res$diag$df_method,
    Warning   = res$diag$warning
  )
}
simple_effects <- dplyr::bind_rows(simple_list)
readr::write_csv(simple_effects, "Swim_vs_Crawl_simple_effects_by_region.csv")
dir.create("plots_swim_vs_crawl", showWarnings = FALSE)
purrr::iwalk(plot_list, ~ ggsave(
  filename = file.path("plots_swim_vs_crawl", paste0(gsub("[^A-Za-z0-9_]+","_", .y), ".png")),
  plot = .x, width = 6, height = 4, dpi = 300
))
if (nrow(simple_effects) > 0) {
  openxlsx::addWorksheet(wb, "SimpleEffects_SwimVsCrawl")
  openxlsx::writeData(wb, "SimpleEffects_SwimVsCrawl", simple_effects)
}
if (length(all_outlier_logs) > 0) {
  openxlsx::addWorksheet(wb, "Outlier_Log_PER_DEP")
  openxlsx::writeData(wb, "Outlier_Log_PER_DEP", dplyr::bind_rows(all_outlier_logs))
}
if (length(all_iqr_stats) > 0) {
  openxlsx::addWorksheet(wb, "IQR_Stats_PER_DEP")
  openxlsx::writeData(wb, "IQR_Stats_PER_DEP", dplyr::bind_rows(all_iqr_stats))
}
if (length(all_model_rows) > 0) {
  openxlsx::addWorksheet(wb, "Model_FixedEffects_RAW")
  openxlsx::writeData(wb, "Model_FixedEffects_RAW", dplyr::bind_rows(all_model_rows))
}
if (length(all_diag_rows) > 0) {
  openxlsx::addWorksheet(wb, "Model_Diagnostics")
  openxlsx::writeData(wb, "Model_Diagnostics", dplyr::bind_rows(all_diag_rows))
}
openxlsx::saveWorkbook(wb, OUTPUT_XLSX, overwrite = TRUE)
cat(sprintf("[OK] Wrote report: %s\n", normalizePath(OUTPUT_XLSX)))
```

Biology Open • Supplementary information

Dataset 1.

Available for download at
https://journals.biologists.com/bio/article-lookup/doi/10.1242/bio.062371#supplementary-data

Dataset 2.

Available for download at
https://journals.biologists.com/bio/article-lookup/doi/10.1242/bio.062371#supplementary-data
